# Carbapenem-Resistant *Klebsiella pneumoniae*: Virulence Factors, Molecular Epidemiology and Latest Updates in Treatment Options

**DOI:** 10.3390/antibiotics12020234

**Published:** 2023-01-21

**Authors:** Theodoros Karampatakis, Katerina Tsergouli, Payam Behzadi

**Affiliations:** 1 Microbiology Department, Papanikolaou General Hospital, 57010 Thessaloniki, Greece; 2 Microbiology Department, Agios Pavlos General Hospital, 55134 Thessaloniki, Greece; 3 Department of Microbiology, Shahr-e-Qods Branch, Islamic Azad University, Tehran 37541-374, Iran

**Keywords:** carbapenem-resistant *Klebsiella pneumoniae*, molecular epidemiology, antimicrobial agents, virulence factors

## Abstract

*Klebsiella pneumoniae* is a Gram-negative opportunistic pathogen responsible for a variety of community and hospital infections. Infections caused by carbapenem-resistant *K. pneumoniae* (CRKP) constitute a major threat for public health and are strongly associated with high rates of mortality, especially in immunocompromised and critically ill patients. Adhesive fimbriae, capsule, lipopolysaccharide (LPS), and siderophores or iron carriers constitute the main virulence factors which contribute to the pathogenicity of *K. pneumoniae*. Colistin and tigecycline constitute some of the last resorts for the treatment of CRKP infections. Carbapenemase production, especially *K. pneumoniae* carbapenemase (KPC) and metallo-β-lactamase (MBL), constitutes the basic molecular mechanism of CRKP emergence. Knowledge of the mechanism of CRKP appearance is crucial, as it can determine the selection of the most suitable antimicrobial agent among those most recently launched. Plazomicin, eravacycline, cefiderocol, temocillin, ceftolozane–tazobactam, imipenem–cilastatin/relebactam, meropenem–vaborbactam, ceftazidime–avibactam and aztreonam–avibactam constitute potent alternatives for treating CRKP infections. The aim of the current review is to highlight the virulence factors and molecular pathogenesis of CRKP and provide recent updates on the molecular epidemiology and antimicrobial treatment options.

## 1. Introduction

*Klebsiella pneumoniae* is a non-motile Gram-negative opportunistic pathogen responsible for approximately 10% of nosocomial bacterial infections. Infections caused by carbapenem-resistant *K. pneumoniae* (CRKP) isolates are a major threat for public health. Such infections can increase the mortality rates of critically ill and debilitated patients hospitalised in intensive care units (ICUs) and can have a negative impact on the financial costs of their hospitalisation all over the world [1,2,3,4]. Remarkably, the mortality rate among patients with pneumonia caused by *K. pneumoniae* is about 50% [5]. Another major topic for public health is the effect of CRKP infections in disability-adjusted-life-years (DALYs) per 100,000 population, with a median of 11.5 in the European Union, and Greece being among the countries with the highest numbers [6]. The rate of carbapenem resistance for *K. pneumoniae* isolates reached 66.3% in 2020 in Greece [7]. A recent meta-analysis shows that the prevalence of CRKP colonisation ranges worldwide from 0.13 to 22% with a pooled prevalence of 5.43%, while the incidence of CRKP colonisation ranges from 2% to 73% with a pooled incidence of 22.3% [8]. CRKP isolates are usually classified as multidrug-resistant (MDR), extensively drug-resistant (XDR) and pandrug-resistant (PDR), which cause even more difficulty in treating infections. According to the European Center for Disease Prevention and Control (ECDC), MDR is defined as ‘acquired non-susceptibility to at least one agent in ≥ three antimicrobial categories, XDR is defined as ‘non-susceptibility to at least one agent in all but ≤ two antimicrobial categories (i.e., bacterial isolates remain susceptible to only one or two categories)’ and PDR is defined as ‘non-susceptibility to all agents in all antimicrobial categories’ [9]. The molecular epidemiology of CRKP isolates is significant as it can determine potential treatment options [10].

The aim of the current review is to highlight the virulence factors and molecular pathogenesis of CRKP and provide recent updates on the molecular epidemiology and antimicrobial treatment options.

## 2. Genomic Pool

Despite the unclear reasons for the high frequency of infections caused by *K. pneumoniae* compared to other Gram-negative opportunistic bacterial pathogens, there are some suggestions comprising genetic element exchanges with human microbiome populations through DNA molecules, mobile genetic element exchanges bearing genes associated with virulence enhancers and antimicrobial resistance, inherent antimicrobial resistance, starvation tolerance and surpassing other bacterial competitors, which may explain the occurrence of this feature [11,12,13,14,15].

According to genomic investigations, the pan-genome of *K. pneumoniae* involves a size of about five to six Mbp bearing five to six kilogenes to be encoded. From this number of encodable genes, about seventeen hundred genes are recognized as core genes. The core genome is conserved among bacterial species of *K. pneumoniae*. Typically, the core genes are present in ≥95% of the members pertaining to a given species. However, the rest genomic pool includes accessory genes. In other words, the accessory genome is known as dispensable, flexible, adaptive or supplementary genome, which varies among *Klebsiella* spp. The accessory genes are typically present in <95% of the members pertaining to a given species [16,17,18].

Indeed, progression and development in microbial taxonomic approaches provides easier diagnostic and detective methodologies in association with epidemiological studies, public health surveillance and outbreak investigations. Due to this knowledge, effective approaches such as core genome multilocus sequence typing (cgMLST) can be recruited for new advanced techniques, including dual barcoding approach [19,20,21].

The *K. pneumoniae* species complex based on genomic phylogenetic structure is categorized into seven major phylogroups comprising Kp1 (*K. pneumoniae subspecies pneumoniae* or *K. pneumoniae sensu stricto*), Kp2 (*K. quasipneumoniae* subsp. *quasipneumoniae*), Kp3 (*K. variicola subsp. variicola*), Kp4 (*K. quasipneumoniae subsp. similipneumoniae*), Kp5 (*K. variicola subsp.* tropica), Kp6 (*K. quasivariicola*) and Kp7 (*K. africana*) [17,19]. In this regard, seven housekeeping genes including *gap*A, *inf*B, *mdh*, *pgi*, *pho*E, *rpo*B and *ton*B are sequenced. Moreover, the K-typing or capsule typing can be achieved through *wzi* gene sequencing or serotyping methods [11].

So, through the MLST typing of the above seven housekeeping genes, several phylo-genetic lineages, e.g., clonal groups (CGs) and/or sequence types, exist [22].

As mentioned above, the antimicrobial-resistant and hypervirulent strains of *K. pneumoniae* have raised great concern worldwide. On the other hand, *Klebsiella* spp. are known as significant bacterial agents isolated from patients with ventilator-associated pneumonia (VAP) in ICUs. According to reported results from previous studies, 83% of hospital-acquired pneumonias are associated with VAP [5,23].

Although ß-lactam antimicrobials are known as the first choice for treatment of infections caused by *K. pneumoniae*, the number of ß-lactamase and especially carbapenemase-producing strains considerably increases. Due to this knowledge, the dissemination of ST258 CRKP is a global concern, as ST258 strains are not completely sensitive towards a wide range of antimicrobials comprising aminoglycosides, fluoroquinolones, etc. [24,25,26,27,28,29,30].

In accordance with the latest studies, the clonal complex (CC) of CC258 is known as the main CRKP comprising ST11, ST258, ST340, ST437 and ST512. Moreover, there are a wide range of MDR clonal groups (CGs), e.g., CG101, CG490, CG147, CG307, CG152, CG14/15, CG231, CG43, CG17/20, CG37 and CG29, which are distributed around the world [31,32,33,34].

According to recorded reports, about 7.5% of STs (or >115 STs) pertaining to CPKP strains have been recognized in different global geographical regions. In addition, CG258 is thepredominant global CPKP strain with 43 ST members. Among them, ST258, ST11, ST340, ST437 and ST512 are the most predominant members of CG258 worldwide. ST11 ranks first in America (Latin) and Asia, while ST258 are the predominant CRKP strains in America (Latin and North) and some European countries. The ST340 has been reported in Greece and Brazil, and ST512 has been identified in Israel, Italy and Colombia [35].

The latest studies depict ≥1452 STs associated with *K. pneumoniae,* in which 1119 STs are recognized as known strains while the remaining 333 are detected as novel STs. In addition to CG258, CG15 and ST307 carry a huge range of antimicrobial resistance genes that are globally disseminated and are associated with healthcare infectious diseases and nosocomial outbreaks [22].

## 3. Virulence Factors and Molecular Pathogenesis

In accordance with the latest categorization, *K. pneumoniae* strains are classified into two major pathotypes, including classical *K. pneumoniae* and hypervirulent *K. pneumoniae* (HVKP). Although the classical type is frequent pathogenic agent relating to hospital acquired pneumoniae (HAP), it has limited virulence capability. Furthermore, the classical pathotype easily tends to exchange mobile genetic elements such as plasmids to create MDR strains, while HVKP is recognized as a causative agent of fulminant and invasive diseases and infections in communities. In addition, the HVKP pathotype is capable of bearing plasmids of hypervirulence or carbapenem resistance [36,37,38,39]. Hence, the capability of virulence gene acquisition of CRKP is known as a major means of hypervirulent CRKP strains production [40,41]. According to the latest reports, the main portion of HVKP strains is composed of antibiotic-sensitive populations excluding ampicillin; however, in recent years the number of convergent *K. pneumoniae* strains is promoting. The convergent *K. pneumoniae* strains are recognized as MDR HVKP strains bearing aerobactin synthesis locus (*iuc*) and producing ESBL or carbapenemase enzymes. The convergent *K. pneumoniae* strains may originate either from those hypervirulent strains which obtained an MDR plasmid or from MDR strains which acquired a virulence plasmid [42].

It is necessary to mention that the identified CPKP strains may bear different genes such as *bla*_IMP_, *bla*_KPC_ and *bla*_NDM_, while the *bla*_KPC_-bearing CPKP strains involve the major portion of the isolated cases from clinical samples worldwide [43,44]. As an effective example, *bla*_KPC_ transmission may occur through a wide range of processes including clonal spread, plasmids and mobile small genetic elements such as transposon (e.g., Tn4401) [35]. Indeed, the Tn4401 is a Tn3-based transposon with a length of 10 Kb which is ended via two genes of Tn3 transpoase (*tnp*A) and Tn3 resolvase (*tnp*R), and two insertion se-quences of ISKpn6 and ISKpn7 [35,45]. The *bla*_KPC_ is known as a plasmid-borne gene which can be carried by > 40 plasmids. These plasmids originate from different incompatibility (Inc) groups such as A/C, ColE, FIA, I2, IncFII, L/M, N, P, R, U, W and X. The *bla*_KPC_ carrier plasmids bear a significant number of antimicrobial resistance genes [35,43]. Moreover, K-typing is normally recruited for HVKP categorization. Although the K1 and K2 types are mostly (~70%) belonging to HVKP and may cause invasive infections, some strains of K1 and K2 types do not pertain to HVKP types [5,46,47,48]. K1, K2, K16, K28, K57 and K63 capsule types are recognized among HVKP strains. The typical phenotypic characteristic of K1 and K2 types is the hypermucoviscous exhibition which can be recognized through a viscous string with a length of more than 5 mm on medium agar [5,49].

Indeed, the integrative conjugal elements and giant plasmids are the effective genetic elements which support the high virulence characteristics in HVKP strains [50,51,52]. *K. pneumoniae* encompasses four important and effective virulence factors, e.g., adhesive fimbriae (including type 1 type 3 fimbriae), capsule, lipopolysaccharide (LPS) and siderophores [5,23,53,54,55].

**Figure 1:**
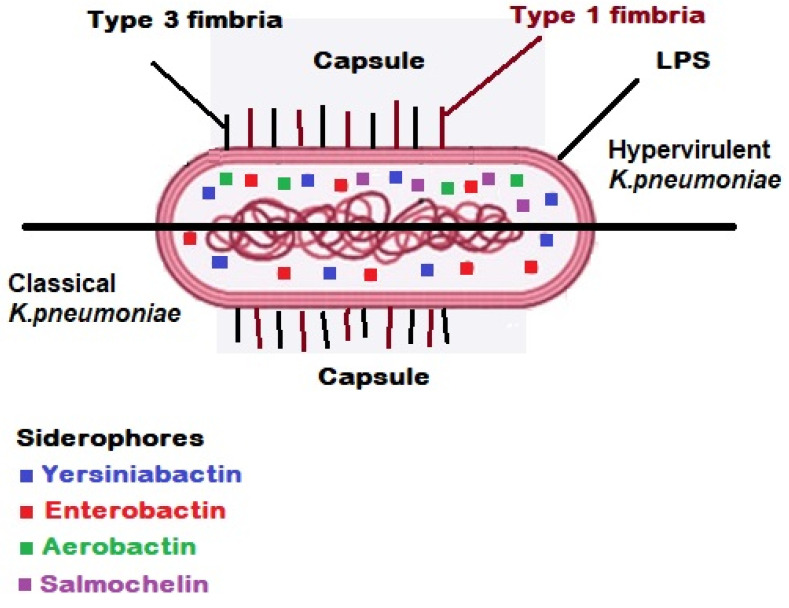
Virulence factors in classical/hypervirulent strains of *K. pneumoniae* [57]. Two types of fimbriae are involved in pathogenesis of the bacteria through attachment to the biotic (human host urothelium) and abiotic (urinary catheter) surfaces to start the process of colonisation, biofilm formation and bacterial invasion. The polysaccharide capsule in *K. pneumoniae* is known as a pivotal virulence factor which acts as the outermost layer in a bacterial cell and interacts with the host. Lipopolysaccharide (LPS) is an effective protective structure against serum complement proteins in parallel with the presence of capsule.

***Adhesive fimbriae:*** *K. pneumoniae* is armed with two important types of fimbriae including type 1 (encoded by fimBEAICDFGH operon) and type 3 (mrkABCDF/mrkABCDEF) fimbriae, which are involved in pathogenesis of the bacteria through attachment to the biotic (human host urothelium) and abiotic (urinary catheter) surfaces to start the process of colonisation, biofilm formation and bacterial invasion (Figure 1) [14,18,56].

***Capsule:*** The polysaccharide capsule in *K. pneumoniae* is known as a pivotal virulence factor which acts as the outermost layer in a bacterial cell and interacts with the host (Figure 1). All types of this acidic polysaccharide capsule are the product of Wzx/Wzy-dependent polymerization pathway encoding by the *cps* gene cluster. The virulence factor of the capsule covers the *K. pneumoniae* bacterial cells against the host immune system responses such as phagocytosis, complement proteins, opsonophagocytosis, oxidative killing and antimicrobial peptides. In another word, the encapsulated bacterial cells of *K. pneumoniae* are capable of evading the host’s immune system through their capsule antigens mimicking the host glycans to survive [27,49,54,55,58,59]. As aforementioned, the K-antigen belonging to *K. pneumoniae* capsule is an effective criterion for classification and serotyping of the pathogenic strain of *K. pneumoniae*. Indeed, sequencing of six genes comprising *galF*, *orf2* (*cpsACP*), *wzi*, *wza*, *wzb* and *wzc* located at the 5′ end of the *cps* gene cluster has shown that these genes are highly conserved, while the mid zone of the *cps* loci encompasses a variable region of nucleotide sequences producing proteins which participate in assembly and polymerization of capsule blocks. Due to this fact, the K-typing method is considered an effective categorization technique. Up to now, >80 serotypes are recognized among pathogenic strains of *K. pneumoniae* according to K-antigen capsule [49,55,58,60,61]. However, up to 70% of *K. pneumoniae* isolated bacterial cells are able to produce a novel capsule or not capable to express any capsule. Hence, this portion of *K. pneumoniae* strains are not typeable through serological methods. Instead, through the contribution of molecular techniques and sequencing technologies, we are able today to investigate the capsule synthesis loci or K-loci belonging to more than 2500 whole genomes of *K. pneumoniae*. The recorded results from previous investigations show 134 distinct K-loci encoding minimally 134 different K-types which can be effective in epidemiological studies in association with *K. pneumoniae* [62]. Capsule is involved in bacterial biofilm formation; the results reported from previous studies depict that unencapsulated strains of *K. pneumoniae* are highly sensitive to host immune responses. Furthermore, the unencapsulated strains of *K. pneumoniae* show reduction in their pathogenicity in mice models [23,54].

The gene clusters encoding capsule are located on chromosome or plasmids. In this regard, the *wzy-K1*, *wzx*, *wzc*, *wza*, *wzb*, *wzi*, *gnd*, *wca*, *cps*A, *cps*B, *cps*G and *gal*F encode exopolysaccharide portion of capsule and are located on chromosome (*wza*, *wzb*, *wzc*, *gnd*, *wca*, *cps*A, *cps*B, *cps*G and *gal*F constitute the cps chromosomal operon gene), while the *rmp*A, *rmp*B and *rmp*A2 genes involved in capsule biosynthesis are locatedon both chromosome and plasmid. Moreover, the genes of *kvr*A, *kvr*B, *rcs*A, *rcs*B, c-*rmp*A and *c-rmp*A2 contribute to capsule biosynthesis and are situated on chromosome. Finally, the genes of *p-rmp*A and *p-rmp*A2, which participate in capsule biosynthesis, are plasmid-borne. *C-rmp*A, *c-rmp*A2, *p-rmp*A and *p-rmp*A2 and *wzy-K1* positively regulate the process of hypercapsulation through affecting the transcripts producing via cps chromosomal operon gene. *Kvr*A, *kvr*B and *rcs*B genes regulate the capsule production through controlling effect on rmpA promoter. Indeed, *rmp*A and *rmp*A2 regulate the mucoidal property in *K. pneumoniae* [54].

***Lipopolysaccharide (LPS):*** LPS is a Gram-negative bacterial endotoxin which is composed of lipid A, O-antigen and an oligosaccharide core. Each constructive part of LPS is respectively encoded by *lpx*, *wbb* and *waa* gene clusters. LPS is an effective protective structure against serum complement proteins in parallel with the presence of capsule (Figure 1). LPS is also a bacterial protector in opposition to the human host humoral immune system. Furthermore, LPS is known as an important inducer biomolecule for toll-like receptor 4 (TLR4), which may activate the expression and secretion of different cytokines and interleukins [23,54,63,64,65,66,67].

***Siderophores or iron carriers:*** The pivotal role of iron related to virulence and pathogenesis of pathogenic microorganisms has been detected. In this regard, there are effective interactions between the iron metabolism and immune cells which affect the pathogenesis of microbial agents (Figure 1) [68,69,70]. Iron molecules are recognized as competitive resources for pathogenic bacteria, e.g., *K. pneumoniae* survival within their host during a successful infection. Therefore, acquiring and recruiting host iron metals by the pathogenic bacteria is an effective strategy to survive and establish infection within the host in the presence of immune cells, e.g., macrophages (MΦs and neutrophils) and molecules. Indeed, as a first line defensive mechanism in a healthy human host immune system, the iron molecules are normally not free within the plasma. To protect the host from the virulence of pathogenic bacterial cells of *K. pneumoniae*, the iron metals are linked to iron transporters of transferrins and iron-binding immunoglycoproteins of lactoferrins [23,68,70,71,72,73].

Iron as an essential element is necessary for both human and microbial pathogens. Iron contributes to different biological features including DNA biosynthesis or replication, transcription, production of energy within mitochondria, central metabolism and enzymatic reactions [73,74]. Hence, the human host body has iron-chelating proteins to bind the iron metals while the pathogens encompass siderophores or iron carriers which bind to iron metal with high affinity. Interestingly, bacterial iron binding proteins are effective competitors to human host iron-chelating proteins. Some bacterial pathogens such as *K. pneumoniae* possess stealth iron carriers. Up to now, several iron scavengers known as siderophores have been recognized among Gram-negative microbial pathogens including enterobactin, aerobactin, yersinobactin, salmochelin, etc., with different levels of affinity for iron molecules. However, *K. pneumoniae* is able to recruit these four iron carriers. According to previous reported results, enterobactin as a highly conserved iron scavenger is the most common siderophore secreted by ~90% of isolated Enterobacterales members. Among the aforementioned iron carriers, enterobactin (encoded by entABCDEF gene cluster upon the chromosome and transported via fepABCDG) has the strongest affinity for iron molecules [54,73,75,76,77,78].

## 4. Mechanisms of Antimicrobial Resistance

*K. pneumoniae* isolates present resistance to antimicrobial agents through one or more of the following mechanisms:

(a) production of specified enzymes (e.g., β-lactamases or aminoglycoside modifying enzymes) [79,80].
(b) decreased cell permeability through loss of Omps [81].
(c) overexpression of efflux pumps, which are transmembrane proteins, with the antimicrobial agent being usually excreted out of the bacterial cell through an energy-consuming process. For example, an efflux pump called KpnGH contributes to antimicrobial resistance in *K. pneumoniae* [82].
(d) modification of the target of the antimicrobial agent [83].

### 4.1. B-Lactams—Ambler Classification of β-Lactamases

B-lactam antimicrobials contain a β-lactam ring in their chemical structure. In this group, the following antimicrobials are classified: (a) penicillin and its derivatives (semisynthetic penicillins), (b) cephalosporins and cephamycins, (c) monobactams and (d) carbapenems (imipenem, meropenem, ertapenem and doripenem). B-lactamases are enzymes that hydrolyse the β-lactam ring, inhibiting the action of these antimicrobials [84].

There are two classification schemes of β-lactamases. Initially, according to the initial functional classification system proposed by Bush, β-lactamases are classified in three major groups, based on their substrate and inhibitor profiles. These functional attributes have been associated with molecular structure in a dendrogram for those enzymes with known amino acid sequences [85].

However, the revised molecular classification proposed by Ambler is the most widely used. Based on this classification, only amino acid sequence determination could provide information upon which a molecular phylogeny could be based. According to preliminary data, β-lactamases have a polyphyletic origin. Thus, they are classified in four different classes, designated A, B, C and D [86,87].

Class A β-lactamases are serine-based enzymes. This class includes simple β-lactamases, such as sylfhydryl variable (SHV), temoneira (TEM), cefotaxime hydrolysing capabilities (CTX-M), *Pseudomonas* extended-resistant (PER), Guiana extended-spectrum (GES), Vietnamese extended-spectrum β-lactamase (VEB), integron-borne cephalosporinase (IBC), Serratia fonticola (SFO), Brazil extended-spectrum (BES), Belgium extended-spectrum (BEL) and Tlahuicas Indians (TLA). All these β-lactamases are inhibited both in vivo and in vitro by β-lactamase inhibitors (clavulanate, tazobactam, sulbactam). SHV and TEM can act, due to point mutations, as extended spectrum β-lactamases (ESBLs), while CTX-M is considered the newest ESBL. All the rest could act as ESBLs with milder hydrolytic capacity. ESBLs can potentially be inhibited by clavulanate, but they have an in vivo therapeutic effect only for urinary tract infections (UTIs). Inhibitor-resistant TEMs (IRTs) and inhibitor-resistant SHVs (IRSs), as well as carbapenemases called *K. pneumoniae* carbapenemases (KPCs), are classified in this group [88,89]. KPCs are distinguished in 12 subtypes [90].

Class B β-lactamases include carbapenemases which are called metallo-β-lactamases (MBLs). Their action is based on zinc ions (Zn^+2^). MBLs hydrolyse all β-lactams except aztreonam, which belongs to monobactams. The most well-known MBLs detected so far are Imipenemase (IMP), Verona integron-encoded MBL (VIM), German imipenemase (GIM), Sao Paulo MBL (SPM), Seoul imipenemase (SIM), Australia imipenemase (AIM), Dutch imipenemase (DIM), New-Delhi MBL (NDM), and the recently detected Tripoli MBL (TMB) and Florence imipenemase (FIM) [91,92,93,94,95,96,97,98,99]. MBLs are classified further in three subgroups: B1, B2 and B3 [87].

Class C β-lactamases include serine-based enzymes, called cephalosporinases or AmpC β-lactamases. They are distinguished as stable and inducible, and they can be either chromosomally or plasmid-located (AmpC-like). The production of inducible AmpC depends on whether the inducer is weak or strong. They are not inhibited by β-lactamase inhibitors, and they are sensitive to cefepime and carbapenems. *K. pneumoniae* strains mainly transfer AmpC-like enzymes, which are considered to have been transmitted from a bacterial chromosome through plasmid conjugation. AmpC β-lactamases are distinguished in various classes [100].

Class D β-lactamases include serine-based enzymes which are called oxacillinases (OXA). These enzymes are characterized by high heterogeneity regarding their structure and their biochemical characteristics. Therefore, they display a large variety concerning their hydrolytic potential depending on the subtype they belong. They are not inhibited by β-lactamase inhibitors. Some of them act as carbapenemases with a milder hydrolytic capacity compared to carbapenemases belonging to other classes. However, they can provide a high grade of resistance when they co-exist with other resistance mechanisms [101].

### 4.2. Decreased Cell Permeability through Loss of Omps

The contribution of OMP deficiency is considered a secondary mechanism conferring mainly a low level of resistance itself. *OmpA*, *OmpK35*, *OmpK36* and *OmpK37* are the most important OMPs in *K. pneumoniae* strains, with a global concern [102].

*OmpA* alterations confer resistance to antimicrobial agents, but not to carbapenems [103]. The mutations of *OmpK35* in combination with these of *OmpK36* usually act as a supplementary mechanism of resistance in the emergence of CRKP isolates [104,105]. The downregulation of *OmpK37* has a minor contribution to the appearance of CRKP [106].

### 4.3. Transport of Antimicrobial Resistance Genes

The antimicrobial resistance genes are encompassed in mobile elements such as plasmids, transposons and integrons. These elements are crucially important, as they are involved in the vertical transmission of these genes from *K. pneumoniae* to its descendants, as well as in the horizontal transmission of the genes from a certain *K. pneumoniae* strain to another.

Most plasmids are usually circular double-stranded DNA molecules, but linear plasmids are also detected. The conjugative plasmids are crucial in the transport of antimicrobial resistance genes from a specific *K. pneumoniae* strain to another and they encode all the appropriate factors for this transfer [107]. There is a strong correlation between specific antimicrobial resistance genes and their integration in certain plasmids. Several of them can transfer many copies of these resistance genes, providing even higher grade of resistance [108]. Transposons are small DNA fragments. They are transported from one DNA site to another but do not have the ability of self-replication. The transfer can be conducted either through transposon duplicate and transport of the copy or through cut and transfer of the whole transposon [109].

Integrons are larger genetic elements which can encompass antimicrobial resistance cassettes and are classified in five classes [110]. They can also be incorporated in other mobile genetic elements such as transposons and conjugative plasmids [111].

## 5. Trends in Molecular Epidemiology

The first MBL detected in a CRKP isolate was IMP-1 in 1996 in Singapore [112]. Since then, CRKP isolates producing IMP have been isolated globally, but mainly in south and southeastern Asia [79,113,114]. VIM MBLs are the most prevalent on a global level. In 2004, an outbreak caused by VIM-1 producing CRKP strains took place in France, after the hospitalisation of a patient in Greece [115]. Since then, several VIM subtypes have been identified, such as VIM-12, VIM-19, VIM-4, VIM-27, VIM-26 and VIM-39, especially in endemic countries for CRKP. These VIM variants are genetically related between them, and they can emerge one from another due to minor genetic events, such as point mutations. The *bla*_VIM_ genes are commonly integrated in a class 1 integron [116]. The ST147 according to the Institut Pasteur scheme has been the most frequently detected among VIM-producing *K. pneumoniae* isolates [117,118]. NDM MBL is a very virulent carbapenemase, as it has a huge capacity to penetrate within the community [119]. A possible explanation could be the presence of a community pool contributing to autochthonous acquisition [120]. It was initially detected in a CRKP isolate in Sweden from the clinical specimen of a patient previously hospitalised in New Delhi, India [121]. Since then, it has disseminated globally and constitutes a threat of major concern [122]. Since its first emergence, ST11 has been the predominant type among NDM producers [123]. All other MBLs are isolated mainly in specific areas and show minor epidemiological concern [91,94,95,96,97,98,99].

KPC is the most prevalent of all carbapenemases. KPC-1 was initially isolated in the United States of America in 1996 and has expanded rapidly to the east coast. It is considered endemic in many parts of New York [90]. However, the major spread of KPC-producing CRKP began in 2007 after an outbreak of CRKP isolates producing KPC-2 in Crete, Greece. These isolates displayed clonal expansion and they were found to be clonally related with the clone of New York which was previously described [124,125]. Since then, this clone has predominated and was named ‘hyperepidemic Greek clone’ [126]. According to the Center for Disease Control and Prevention (CDC), around 70% of KPC-2 producing CRKP isolates are assigned to ST258 [127]. ST258 has been associated with multidrug resistance to antimicrobials [128]. However, ST258 KPC-2 producing CRKP isolates are considered low-virulent and are opportunistic pathogens, as only a low proportion of patients colonised with these isolates develop an infection. These CRKP isolates create extended reservoirs with the virulence and mortality rates being relatively low [129]. Patients with co-morbidities and chronic diseases are more vulnerable in suffering from an infection [130]. Recently, KPC-2 CRKP belonging to ST39 have emerged [131].

CRKP isolates harbouring concurrently VIM-1 and KPC-2 are usually assigned in ST147, meaning that they are commonly related with VIM-1 [132]. However, CRKP isolates producing concurrently VIM-1 and KPC-2 have recently been assigned to ST39, implying some kind of relatedness with KPC-producers [133].

Regarding class D carbapenemases, the most prevalent carbapenemase is OXA-48, initially detected in Turkey in 2001. OXA-48 hydrolyses carbapenems in a mild way, conferring a low level of resistance and its action is accompanied with additional resistance mechanisms. Since 2007, OXA-48 producing CRKP isolates have been detected in many countries in Europe and north Africa. However, these isolates are not considered highly virulent. ST11 is the most prevalent among OXA-48 producers [79,134,135]. OXA-162, initially detected in Turkey is a variant of OXA-48, as well as OXA-181 which is the second most prevalent OXA detected worldwide [79,136]. One of the latest OXA subtypes detected is an OXA-48 variant, designated OXA-370, isolated in Brazil in 2014 [137].

The contribution of Omp loss to CRKP emergence is trivial. Omp loss is usually a secondary mechanism which provides low levels of resistance to antimicrobial agents and can act along with carbapenemase action. The most significant Omps in *K. pneumoniae* isolates are *OmpA, OmpK35, OmpK36* and *OmpK37* [102]. Changes in *OmpA* are generally associated with antimicrobial resistance, but not with CRKP appearance [103]. The role of *OmpK35* in carbapenem resistance has been highlighted since 2003, and the contribution of *OmpK36* around 2005 [81,138]. Since then, there has been global concern concerning the role of point mutations in genes encoding *OmpK35* and *OmpK36* as a complementary mechanism in the emergence of CRKP [104,105]. However, in 2012 an outbreak which took place in Greece led to the emergence of clonally related CRKP isolates with resistance to ertapenem exclusively due to down-regulation of *OmpK35* and mutated *OmpK36* [139]. The reduced expression of *OmpK37* has not been associated with the emergence of CRKP [106].

## 6. Trends in Antimicrobial Treatment

### 6.1. Colistin

Colistin is an antimicrobial which was discovered in 1949 and belongs to polymyxins (polymyxin E) (Table 1). Its use was abandoned at the beginning of the 1980s due to the high nephrotoxicity observed during its administration [140]. However, due to the spread of antimicrobial resistance and the appearance of CRKP and MDR *K. pneumoniae* (MDRKP), it has revived and is used as first-line treatment for infections caused by these isolates [141].

Colistin has been used widely for the treatment of VAP, bacteremias, abdominal infections and UTIs caused by CRKP and MDRKP [10]. However, the implementation of colistin monotherapy against these infections has been associated with a negative outcome, the emergence of antimicrobial resistance with the emergence of colistin-resistant CRKP [142,143]. Therefore, colistin is usually administered in combined therapeutic protocols along with tigecycline or aminoglycosides, in triple combined schemes along with tigecycline and carbapenems, fosfomycin or aminoglycosides, and in quadruple treatment schemes [1]. In CRKP isolates with a relatively low grade of resistance to carbapenems, therapeutic schemes combining colistin with carbapenems seem to be more effective, while in CRKP isolates with a high grade of carbapenem-resistance, therapeutic regimens including colistin and high dosages of tigecycline, fosfomycin and aminoglycosides present more satisfying results [144]. The advance of knowledge around the dosage of intravenous colistin administration and the progress in the pharmacokinetics of colistin have led to more satisfying therapeutic effects and reduced nephrotoxicity [10,145] As far as the treatment of infections caused by colistin-resistant CRKP isolates is concerned, the combination of colistin with carbapenems of rifampicin has been proven a possible option during the previous years [146,147].

However, it is worth mentioning that despite the fact that colistin is widely used in real-world practice, it is not considered a first-line agent for the treatment of CRKP infections [148,149].

### 6.2. Tigecycline

Tigecycline is a derivative of minocycline and belongs to glycylcyclines. It has a broad spectrum of action and has been used for the treatment of CRKP infections achieving high concentrations in various biological fluids such as lung, skin, soft tissues and bones [150]. When combined with colistin, it presents bactericidal action against CRKP isolates (Table 1) [142,150,151].

### 6.3. Fosfomycin

Fosfomycin is an old antimicrobial agent which has been re-introduced for the treatment of uncomplicated CRKP UTIs (Table 1) [152]. When combined with colistin, its bacterial killing efficacy is greater against CRKP [153].

Over the last five years, several antimicrobials with various activity against MDR Gram-negative bacteria have been launched and approved by the U.S. Food and Drug Administration (FDA) and the European Medical Agency (EMA). These drugs are plazomicin, eravacycline, cefiderocol and temocillin, a β-lactam which has only been approved in Belgium and the United Kingdom. Moreover, ceftolozane–tazobactam, meropenem–vaborbactam, imipenem–cilastatin/relebactam and ceftazidime–avibactam (CAZ-AVI) are antimicrobials that combine β-lactams with β-lactamase inhibitors and are potent alternatives [154].

**Table 1:** General Characteristics of Antimicrobials [155].

Antimicrobial	PubChem CID	Molecular Formula	Synonyms	Structure	Mode of Action
Colistin	44144393	C_52_H_98_N_16_O_13_	Polymyxin E	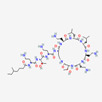	Polycationic peptides which targets bacterial (in particular, Gram-negative bacteria) cell membrane to disrupt it through detergent-like mechanism.
Tigecycline	54686904	C_29_H_39_N_5_O_8_	Tygacil	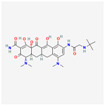	A member of the new class of glycylcyclines. Indeed, glycylcyclines are derived from tetracyclines. Tigecycline targets the ribosomal small subunit of 30S (with higher affinity than tetracyclines) to prevent bacterial protein translation. The attachment of tigecycline to the amino-acyl tRNA molecule inhibits the entrance of the amino-acyl tRNA molecule into the A site of the ribosome to stop the elongation process of the bacterial peptide biosynthesis
Fosfomycin	446987	C_3_H_7_O_4_P	Phosphomycin, Phosphonomycin	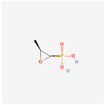	Fosfomycin is used against bacterial strains with the property of difficult-to-treat. This antibiotic is the first option against UTIs. Fosfomycin inactivates the UDP-N-acetylglucosamine enolpyruvyl transferase (MurA) enzyme via binding to a cysteine residue of the enzyme’s active site. This process results in prevention of peptidoglycan precursor UDP N-acetylmuramic acid (UDP-MurNAc) biosynthesis and in consequence may lead to stopping bacterial cell wall biosynthesis. Therefore, fosfomycin has a bactericidal effect on pathogens.
Plazomicin	42613186	C_25_H_48_N_6_O_10_	Zemdri	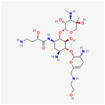	Plazomicin, as a member of aminoglycoside antibiotics, has bactericidal effect through binding to ribosomal small subunit of 30S. This antibiotic changes the spatial structure of the ribosomal A-site (aminoacyl-tRNA site), which may lead to attachment of antibiotic to rRNA molecule. This feature results in mistranslation of mRNA molecules within the process of protein biosynthesis.
Eravacycline	54726192	C_27_H_31_FN_4_O_8_	Xerava	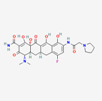	Eravacycline as a fluorocycline antibacterial pertaining to tetracycline class has disruptive effect on bacterial pathogens through targeting their protein biosynthesis processes. This effect is achieved via targeting the ribosomal small subunit of 30S.
Cefiderocol	77843966	C_30_H_34_ClN_7_O_10_S_2_	SZ34OMG6E8	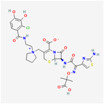	As a cephalosporin drug, has bactericidal effect on aerobic Gram-negative bacteria such as *K.pneumoniae*. Cefiderocol binds to penicillin-binding proteins (PBPs) (in particular with PBP3 and in general with PBP1a, PBP1b, PBP2 and PBP4), inactivating their activities which may lead to inhibition of bacterial cell wall biosynthesis.
Temocillin	171758	C_16_H_18_N_2_O_7_S_2_	Negaban	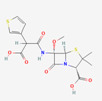	Temocillin acts as inhibitor against bacterial reproduction and growth processes.
Ceftolozan–-tazobactam	86291594	C_33_H_42_N_16_O_13_S_3_	Zerbaxa	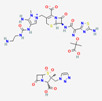	Zerbaxa prevents the growth and reproduction process in bacterial pathogens and has bactericidal effect on UTIs’ bacterial causative agents.
Imipenem–cilastatin	17756656	C_28_H_43_N_5_O_9_S_2_	Thienam	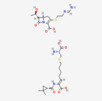	Imipenem–cilastatin prevents the growth and reproduction process in bacterial pathogens. Thienam prevents/antagonizes the process of biosynthesis/actions of the enzymes of bacterial proteases.
Meropenem–vaborbactam	86298703	C_29_H_41_BN_4_O_10_S_2_	Carbavance	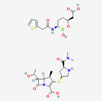	Vaborbactam acts as bacterial serine-ß-lactamases to support the antibacterial effect of penem drugs, e.g., meropenem against CRCKP strains of *K.pneumoniae*, etc.
Ceftazidime–avibactam	90643431	C_29_H_33_N_9_O_13_S_3_	Avycaz	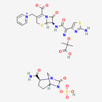	Avycaz prevents the growth and reproduction process in bacterial pathogens. Avibactam prevents/blocks the activities of bacterial ß-lactamases.

### 6.4. Plazomicin

Plazomicin is a synthetic aminoglycoside which was approved in 2018 for the treatment of complicated UTIs (cUTIs) and pyelonephritis [154]. Plazomicin is effective against CRKP and has been correlated with excellent activity against KPC-producing isolates (92.9%), and OXA-48 isolates (87.0%). However, its action against MBL-producing isolates is limited (40.5%) (Table 1) [156].

### 6.5. Eravacycline

Eravacycline is a fluorocycline which is two- to fourfold times more active than tigecycline (Table 1) [157]. Eravacycline is active against carbapenem-resistant Gram-negative bacteria (Johnston et al., 2020) and moreover, against a major proportion of NDM- and VIM-producing CRKP (61.3% and 66.7%, respectively) [158]. However, appearance of eravacycline resistance among CRKP due to over-expression of efflux pumps has already occurred [159].

### 6.6. Cefiderocol

Cefiderocol is a catechol-substituted siderophore (Table 1). It has been approved by the FDA for the treatment of cUTIs in 2019 and for the treatment of ventilator-associated pneumonia (VAP) in 2020 [160]. Cefiderocol inhibits the overwhelming majority of MDR Gram-negative bacteria and is active against CRKP isolates, independently of the existing resistance mechanism [161]. Resistance to cefiderocol has already emerged, especially in MBL-producing CRKP isolates [162]. In addition, co-resistance to cefiderocol and other antimicrobials in KPC-producing CRKP has already been described [163].

### 6.7. Temocillin

Temocillin is 6-α-methoxy derivative of ticarcillin. Some initial pharmacokinetic properties have displayed some action against KPC-producing CRKP causing UTIs (Table 1) [164]. However, the susceptibility of temocillin against KPC-producing CRKP causing UTIs varies among studies. A study in Poland shows 0% susceptibility, while another one in Greece displays only 8.6% [165,166]. However, a study performed in the UK displays an increased susceptibility of 50.8% [167]. In addition, it is not active against MBL- and OXA-48 producing CRKP isolates [166].

### 6.8. Ceftolozane–Tazobactam

Ceftolozane–tazobactam is a combination of β-lactam with b-lactamase inhibitor which was approved by the FDA in 2014 for the treatment of cUTIs and intra-abdominal infections (IAI) (Table 1). Furthermore, the approval was extended for VAP in 2019 [154]. However, ceftolozane–tazobactam is mainly active against *K. pneumoniae* isolates producing ESBL, but not against CRKP strains [168].

### 6.9. Imipenem–Cilastatin/Relebactam

Imipenem–cilastatin/relebactam was approved in 2019 by the FDA for the treatment of cUTIs and IAIs (Table 1). In 2020, it obtained approval for VAP [154]. Relebactam inhibits class A and C β-lactamases. Therefore, it is active against KPC-producing CRKP isolates. However, the addition of relebactam does not restore the activity of imipenem against MBL-producing CRKP [169]. In addition, it does not inhibit adequately OXA-48 producing CRKP [170]. However, resistance to this agent has recently emerged due to genetic rearrangement [171].

### 6.10. Meropenem–Vaborbactam

Vaborbactam is a cyclic boronate derivative (Table 1). When combined with meropenem, it increases the activity of meropenem against KPC-producing CRKP. It has been approved for the treatment of cUTIs, IAIs and VAP [172]. However, it is ineffective against MBL-producing CRKP, while its action against OXA-48 producers is limited [173]. However, resistance to meropenem–vaborbactam has lately appeared because of mutated OmpK35 and OmpK36 [174].

### 6.11. Ceftazidime–Avibactam

Avibactam (formerly NXL104, AVE1330A) was patented in 2011 and is a non-β-lactam β-lactamase inhibitor which is active in vitro against Ambler class A and C β-lactamases and displays some activity against some OXA-type β-lactamases, classified in Ambler class D. Avibactam binds covalently to β-lactamases through the creation of a carbamate bond between avibactam’s position 7 carbonyl carbon and the same active-site serine that participates in acyl bonding with β-lactam substrates (Table 1) [175].

An initial study conducted in China during 2011–2012 has highlighted the in vitro activity of CAZ-AVI against CRKP and other carbapenem-resistant Gram-negative bacteria producing ESBL, AmpC and KPC. These isolates were clinically the cause for IAIs, UTIs, VAPs and bloodstream infections (BSIs) [176]. Some other studies reach the same conclusions [177,178]. It has also been proven to be active against hypervirulent CRKP isolates [179]. CAZ-AVI is normally not active against MBL-producing CRKP. However, it has been combined with aztreonam in the treatment of some cases of NDM-producing CRKP [180,181]. This combination has been applied recently in a patient with complications of SARS-CoV-2 nosocomial infection [182]. Apart from KPC-producing CRKP, CAZ-AVI has been proved effective and safe in vivo against OXA-48 producers [183].

Several clinical randomised control trials (RCTs) have attempted to investigate the efficacy and safety of CAZ-AVI in treating complicated IAIs and UTIs [184,185]. CAZ-AVI was approved by the FDA in the beginning of 2015 for the treatment of cIAIs (combined with metronidazole) and cUTIs at a dose regimen of 2.5 g every eight hours intravenously [186]. The dosing regimens have been later reviewed again in critically ill patients [187]. In addition, CAZ-AVI has been used in many cases as off-label indication or salvage therapy, with promising clinical and microbiological cure rates [188,189].

In addition, the testing of CAZ-AVI against MDR Gram-negative bacteria causing VAP has showed satisfying results [190]. It has been classified as an emerging drug for the treatment of HAP [191]. Since then, a specific RCT has highlighted the efficacy of CAZ-AVI in the treatment of VAP. Based on the results of this study called pivotal phase III REPROVE trial, the FDA approved the use of CAZ-AVI for treatment of patients with HAP/VAP [192].

In addition, some initial attempts of successful treatment of CRKP BSI have been reported [193]. The in vitro activity of CAZ-AVI against CRKP causing BSIs in cancer patients was later revealed [194]. It has also been effective in vivo against CRKP causing BSIs in hematologic patients [195]. Several studies have highlighted CAZ-AVI with higher clinical cure rates and survival than other drugs in treating CRKP BSIs [196].

However, resistance to CAZ-AVI among KPC-producing CRKP has appeared. Some previous studies underline the inability of avibactam to inhibit several KPC-2 variants [197]. Resistance to KPC-3 variant has been also detected early [198,199,200]. In addition, resistance has also emerged due to selective pressure during treatment with CAZ-AVI for a KPC-2 CRKP infection [131].

Resistance to CAZ-AVI seems to have a lesser impact on vaborbactam, implying the use of meropenem–vaborbactam previously described as a potent treatment alternative [201]. Notably, meropenem–vaborbactam has been used successfully in combination with aztreonam for the treatment of ceftazidime-resistant CRKP isolates causing BSIs [202]. Moreover, CAZ-AVI resistance could emerge when administered simultaneously with meropenem–vaborbactam for treating CRKP infections [203]. The appearance of ceftazidime-resistant KPC-producing CRKP is very alarming, as it can cause severe outbreaks in SARS-CoV-2 ICUs [204].

### 6.12. Aztreonam–Avibactam

Aztreonam–avibactam is a combination antimicrobial agent with activity against MBL-producing CRKP. However, it has not been yet approved by FDA [205]. According to recent studies, it is considered an option for the treatment of BSIs caused by colistin-resistant and CAZ-AVI-resistant CRKP isolates [206].

### 6.13. Guidelines for the Treatment of CRKP Infections

According to the latest guidelines of the European Society of Clinical Microbiology and Infectious Diseases (ESCMID) for patients with severe CRKP infections, meropenem-vaborbactam or ceftazidime–avibactam are recommended, if active in vitro. For patients with CRKP infections due to MBL-producing strains, cefiderocol is conditionally recommended. For non-severe CRKP infections, the use of old antimicrobials is advisable depending on the source of infection, while for cUTIs, aminoglycosides including plazomicin are recommended. If necessary, tigecycline could be used in high doses for the treatment of CRKP pneumonia, but not for BSIs and HAP/VAP [148].

The Infectious Diseases Society of America (IDSA) proposes ciprofloxacin, levofloxacin, trimethoprim–sulfamethoxazole, nitrofurantoin, or a single dose of an aminoglycoside for the treatment of uncomplicated cystitis caused by CRKP, and ciprofloxacin, levofloxacin or trimethoprim–sulfamethoxazole for cUTIs. For patients with severe CRKP infection outside the urinary tract system, ceftazidime–avibactam, meropenem–vaborbactam, and imipenem–cilastatin–relebactam are recommended, while for patients with a diagnosed MBL-producing CRKP infection, ceftazidime–avibactam plus aztreonam, or cefiderocol as monotherapy are proposed. Tigecycline and eravacycline are not recommended as monotherapy for the treatment of CRKP UTIs and BSIs, while according to IDSA, colistin should be avoided for the treatment of CRKP infections due to increased mortality and high nephrotoxicity compared to other antimicrobial options [149].

## 7. Conclusions

CRKP infections constitute a significant threat for public health. The knowledge of the exact mechanism of CRKP emergence is crucial for the selection of the most appropriate antimicrobial among those most recently launched. Plazomicin, eravacycline, cefiderocol, temocillin, ceftolozane–tazobactam, imipenem–cilastatin/relebactam, meropenem-vaborbactam, ceftazidime–avibactam and aztreonam–avibactam constitute potent alternatives for treating CRKP infections. The evolution of the molecular epidemiology of CRKP strains is dynamic and data and information around it should be continuously updated to diminish the spread of these isolates.

## Data Availability

No new data were created or analyzed in this study. Data sharing is not applicable to this article.
